# The Effect of the Rotating Disk Geometry on the Flow and Flux Enhancement in a Dynamic Filtration System

**DOI:** 10.3390/membranes13030291

**Published:** 2023-02-28

**Authors:** Jo Eun Park, Tae Gon Kang, Heejang Moon

**Affiliations:** 1 School of Aerospace and Mechanical Engineering, Korea Aerospace University, 76 Hanggongdaehak-ro, Deogyang-gu, Goyang-si 10540, Gyeonggi-do, Republic of Korea; 2 Department of Smart Air Mobility, Korea Aerospace University, 76 Hanggongdaehak-ro, Deogyang-gu, Goyang-si 10540, Gyeongi-do, Republic of Korea

**Keywords:** membrane filtration, dynamic filtration, rotating flow, patterned surface, flux enhancement, numerical simulation

## Abstract

A numerical study was conducted to investigate the effect of rotating patterned disks on the flow and permeate flux in a dynamic filtration (DF) system. The DF system consists of a rotating patterned disk and a stationary housing with a circular flat membrane. The feed flow is driven by the rotating disk with the angular velocity ranging from 200 to 1000 rpm and the applied pressure difference between inlet and outlet ports. Wheel-shaped patterns are engraved on the disk surfaces to add perturbation to the flow field and improve the permeate flux in the filtration system. Five disks with varying numbers of patterns were used in numerical simulations to examine the effects of the number of patterns and the angular velocity of the disk on the flow and permeate flux in the DF system. The flow characteristics are studied using the velocity profiles, the cross-sectional velocity vectors, the vortex structures, and the shear stress distribution. The wheel-shaped patterns shift the central core layer in the circumferential velocity profile towards the membrane, leading to higher shear stresses at the membrane and higher flux compared to a plain disk. When the number of patterns on the disk exceeded eight at a fixed Reynolds number, there were significant increases in wall shear stress and permeate flux compared to a plain disk filtration system with no pattern.

## 1. Introduction

Membrane filtration is a crucial process in a variety of industries, including the biotechnological, chemical, food, and pharmaceutical industries. In pressure-driven membrane filtration processes, several types of membrane modules are used in such industries: tubular modules, flat-sheet modules, hollow fiber modules, and spiral-wound modules [1]. The typical feed flow in such modules is parallel to membrane surfaces and the permeate flow, directed perpendicular to the membrane surface, is produced by the transmembrane pressure difference. Thus, the flow near the membrane surface leads to the accumulation of foulants and the formation of concentration boundary layer near the surface, which are called membrane fouling and concentration polarization, respectively. Fouling is caused by the particles contained in the feed solution that block membrane pores, and creates a cake layer due to deposited particles on the membrane surface. Concentration polarization (CP) refers to a phenomenon in which the species concentration rapidly increases within a boundary layer close to the membrane surface due to the selective transport of species through the membrane [2,3,4,5,6]. Both fouling and concentration polarization leading to a decline in the filtration performance and lifespan of a membrane module are critical issues that should be overcome or alleviated in most filtration processes [7,8,9].

Hydrodynamic control of membrane fouling (or concentration polarization) is an option to minimize fouling and achieve enhanced filtration performance. Flow control in crossflow filtration (CFF) involves either a patterned surface morphology or a static mixer [10,11,12], whereas hydrodynamic control of membrane fouling in spiral-wound modules (SWMs) is usually improved by carefully designed feed spacers [13,14,15,16]. Additionally, an optimized feed channel for a spiral-wound membrane filtration can enhance mass transfer, reduce membrane scaling, and increase flux in the reverse osmosis process [14]. To mitigate fouling or concentration polarization in a filtration system, a rotating disk or rotor blade can be adopted. The flows induced by these rotating parts increase the shear stresses on the membrane surface, promoting back transport of foulants via the rotating flows [17,18,19,20,21,22,23,24]. In addition, a rotating disk over a membrane can effectively enhance mass transfer by promoting mixing near the membrane surface. This type of filtration, which utilizes moving parts to generate a unique flow pattern resulting in enhanced filtration performance, is known as dynamic filtration (DF).

Extensive research has been carried out on DF systems since the late 1980s. Kroner and Nissinen [17] proposed a dynamic filtration module creating Taylor vortices in an annular gap by use of an axially rotating filter. They found that the rotating filtration module resulted in a better performance compared with crossflow filtration modules. Holeschovsky and Cooney [18] developed a CP model for a rotary filtration module with a rotating membrane and a stationary outer cylinder, demonstrating a superior performance of their module compared with crossflow filtration modules. Lee et al. [19] conducted an experimental study on the cell harvest using a rotating disk filtration system. The rotating speed of the disk was found to be the most critical parameter determining shear rates, flow patterns, and the bioseparation performance. The flux increased with the disk rotating speed, but at the expense of additional power input and heat dissipation. Ding et al. [20] investigated the performance of a multishaft disk (MSD) system with ceramic membrane disks mounted on two rotating shafts surrounded by a stationary housing, especially at high speed and with concentrated suspensions. As a sequel to [20], He et al. [21] subsequently conducted an experimental study on a modification of the MSD, where disks of one shaft were replaced by non-permeating metal disks rotating at a different speed to that of the membrane disks. The permeate flux was more influenced by the rotation speed of the membrane disk than that of the metal disks. In addition, disks with vanes resulted in a higher permeate flux and a lower energy consumption compared with those from smooth disks. Recently, Chaudhuri et al. extensively investigated changes of fouling, cake resistance, and concentration polarization for roto-dynamic reverse osmosis filtration systems [22,23,24]. As for a comprehensive review on dynamic filtration, one may refer to two papers by Jaffrin [25,26] and a recent paper by Cheng et al. [27].

From the viewpoint of hydrodynamics in a DF system, the enhanced filtration performance is due to a specific rotating flow pattern in the filtration system [26]. The velocity field near the membrane and the resulting shear stress distribution on the membrane are important factors, with influences on the flux decline caused by membrane fouling or concentration polarization. In this regard, a systematic numerical approach employing computational fluid dynamics (CFD) is widely used in dynamic filtration to obtain the in-depth flow field, the shear stresses, and the solute concentration near the membrane, which are influenced by the disk shape, the disk rotating speed, the operating pressure, etc. [28,29,30,31,32]. The flow in the previous numerical studies belongs to a laminar [29,30] or turbulent flow regime [31,32], depending on the material properties of the feed, the rotating speed of the disk, and the geometrical parameters of a specific dynamic filtration system. The flow regime is described by dimensionless numbers such as the Reynolds number (Re) and the aspect ratio (G) in a rotating system, which will be discussed in Section 2. In this study, our focus is on a rotor-stator-type dynamic filtration system. The system is composed of a rotating patterned disk and a stationary housing with a circular flat membrane.

Rotating-disk systems, on which our filtration system is based, are widely used in various applications such as turbomachinery, computer hard disk drives, automotive brake systems, medical equipment, membrane filtration systems, etc. [33]. The main concern of such applications from the viewpoint of fluid mechanics is the interaction between a spinning disk and the fluid adjacent to the disk, creating complicated three-dimensional rotating flows as observed in a rotor-stator system composed of a spinning disk and a close-fitting stationary housing [34]. Since the pioneering work of Ekman in 1905 [35], tremendous research efforts have been made to understand the in-depth flow characteristics due to a rotating disk [36,37,38,39,40]. Especially with regard to the flow in a closed rotor-stator cavity, Daily and Nece [41] performed experimental and theoretical studies and found the existence of four flow regimes according to Re and G. Chew and co-workers [42,43] conducted an early numerical study on the flow inside a rotor-stator cavity with a centrifugal throughflow. 

In fluids engineering applications, patterned surfaces are used as a means to add perturbations to a flow field or to guide a flow to a desired direction, where the detailed flows are affected by the topology and the geometrical parameters of a surface pattern [44,45,46,47]. In membrane filtration, a large number of studies on patterned solid surfaces or patterned membranes are being conducted to elucidate their influences on antifouling and flux enhancement in crossflow filtration [48,49,50,51,52,53,54]. Flow and mass transfer characteristics due to various engineered surface features [50] and bio-mimetic surface patterns [51] are main concerns of researchers. In particular, the relative size between a surface pattern and a filtration module plays a key role in mitigating membrane fouling or concentration polarization [53,54]. In a DF system, a rotating disk with specific surface structure, such as radial vanes or perforations used in [21,28], can be used to increase the wall shear rate on the membrane and the permeate flux. 

As far as the effect of patterned disks on the flow and filtration performance in dynamic filtration is concerned, however, limited studies have been conducted and further research is needed to advance our understanding on this issue. This study focuses on a rotating-disk type DF system with a wheel-shaped pattern on the disk surfaces. To ensure the effectiveness of the newly designed DF system, it is crucial to understand the hydrodynamics in the filtration system in a wide range of operating conditions as the geometrical parameters of the surface pattern change. For this, computational fluid dynamics (CFD) can be utilized as the virtual product (or process) design tool for a filtration system.

The objectives of the present study are to understand the in-depth flow characteristics and permeate flux enhancement due to the rotating patterned disk in a dynamic filtration system. The focus is on investigating the effects of the number of wheel-shaped patterns and the rotating speed of the disk on the flow and filtration characteristics. The paper is structured as follows. First, the filtration system is described, including the geometries of the filtration module, patterned disks, and operating conditions. Next, the governing equations and relevant boundary conditions to solve the flow problem are presented. The flow characteristics induced by the rotating disk and the applied pressure difference between the inlet and outlet are discussed in detail. The change of vortex structures with the number of patterns is also presented since vortex structures are closely related to the back transport of foulants. Finally, the permeate flux enhancement and shear stress changes, which are highly influenced by the rotating speed of the disk and the number of patterns, are demonstrated.

## 2. Problem Description

### 2.1. Dynamic Filtration Sytem and Operating Conditions

**Figure 1:**
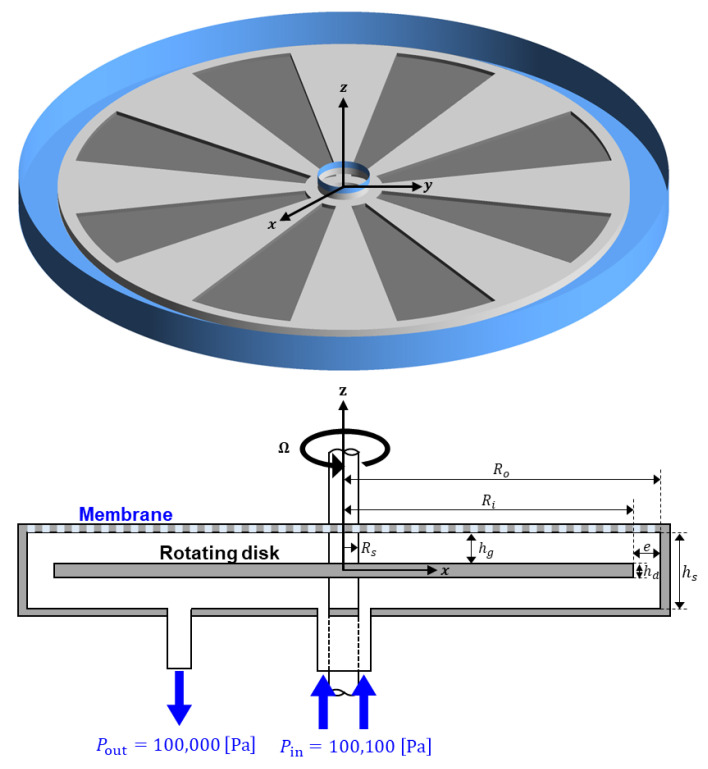
The schematic representation of the dynamic filtration system consisting of a disk (in this figure, with eight engraved patterns) rotating with a constant angular velocity about the z-axis, and a stationary housing. The feed is introduced through an annular-shaped inlet and exits the filtration system through an outlet. A constant pressure difference ($\Delta P_{io}=P_{in}-P_{out}=100$ Pa) is applied between the inlet and outlet. A circular flat membrane with the radius $R_{o}$ is fixed on top of the housing.

Figure 1 schematically represents the dynamic filtration system used in this study, which is a modification of a commercial dynamic microfiltration module, FMX-B (BKT Co. Ltd., Daejeon, Republic of Korea) [28]. The stator radius ($R_{o}$), the disk radius ($R_{i}$), the shaft radius ($R_{s}$), the disk thickness ($h_{d}$), and the axial gap ($h_{g}$) between the disk and the housing are as follows: $R_{o}=100\;\mathrm{mm}$, $R_{i}=88\;\mathrm{mm}$, $R_{s}=8\;\mathrm{mm}$, $h_{d}=3\;\mathrm{mm}$, and $h_{g}=4.5\;\mathrm{mm}$. A circular flat membrane with the radius $R_{o}$ is fixed on top of the housing. A wheel-shaped patterned disk rotates about the z-axis within the stationary housing with the angular velocity Ω, ranging from 200 to 1000 rpm in the present study. As depicted in Figure 1, the feed fluid enters the filtration module through an annular-shaped inlet and exits through an outlet. The pressure at the inlet is $P_{\mathrm{in}}=100,100\;\mathrm{Pa}$ and that at the outlet is $P_{\mathrm{out}}=100,000\;\mathrm{Pa}$. Thus, the pressure difference between the inlet and outlet is fixed at $\Delta P_{io}=100\;\mathrm{Pa}$. The gauge pressure in the permeate side is assumed to be zero. The flow in the filtration system is mostly influenced by the rotational motion of the disk rather than the pressure difference $\Delta P_{io}$ because the scale of the pressure difference in the rotor-stator system ($\rho \Omega^{2}R_{i}^{2}/2$) is much higher than $\Delta P_{io}$ for the operating conditions used in this study.

**Figure 2:**
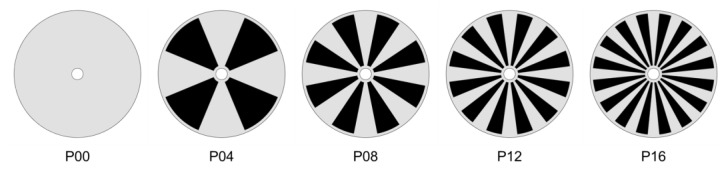
The five disks used in this study with the number of patterns $N_{p}=$ 0, 4, 8, 12, and 16, which are denoted by P00, P04, P08, P12, and P16, respectively. In each patterned disk, engraved areas are filled with the black color. In the cases of patterned disks, the pattern depth is 1.25 mm.

The dynamic filtration system creates high shear stresses at the membrane surface, mainly induced by the mechanical movement of the disk rather than the feed flow rate. The pressure-driven flow due to $\Delta P_{io}$ may add hydrodynamic perturbations to the rotational flow, with the perturbations affected by the inlet flowrate that is slightly higher than the permeate flowrate [21,29,34]. Five disks are used to investigate the effect of the number of patterns $N_{p}$ on the flow and permeate flux at a specific operating condition. Figure 2 schematically depicts the patterns on the circular disk, where wheel-shaped surface patterns (areas filled with the black color) are engraved on both sides of the disk. The pattern depth is fixed to be 1.25 mm. In Figure 2, P00 stands for a plain disk ($N_{p}=0$), P04 for a disk with $N_{p}=4$, P08 for a disk with $N_{p}=8$, etc.

For a given set of the geometrical parameters, fluid properties, and angular velocity of the disk, the rotating flow in a rotor-stator cavity is known to be governed by two dimensionless parameters [41], the aspect ratio G and the Reynolds number Re, defined by

(1) $$G=\frac{h_{g}}{R_{i}}$$

(2) Re=ρΩRi2μ

where ρ and μ denote the density and the viscosity of the feed, respectively. The Reynolds number is defined using the characteristic velocity $R_{i}\Omega$ and the characteristics length $R_{i}$. The feed fluid used in this study has the following density and viscosity: $\rho =997\;\mathrm{kg}/m^{3}$ and μ=0.001 Pa·s. Thus, the two dimensionless parameters are G=0.0511 and 1.6×105≤Re≤8.0×105. When estimated from the diagram for the flow regimes in a rotor-stator cavity flow presented by Daily and Nece [41], the rotating flow caused by the plain disk (P00) belongs to the regime IV, which is characterized by turbulent flow, large clearance, and separate boundary layers. The diagram in [41] serves as the reference to categorize the flow in a rotor-stator-type dynamic filtration system in which the influence of the pressure-driven flow is not significant compared to the flow created by the rotating disk. 

### 2.2. Modeling

If the criterion in [41] is applied to the values of Re and G used in this study, the rotating flow is turbulent with separated boundary layers. The Reynolds-averaged Navier-Stokes (RANS) model [55] was used to describe the turbulent flow. In the case of incompressible Newtonian fluid flows, where the density and dynamic viscosity remain constant, the continuity and momentum equations for the mean velocity components (written in index notation in Cartesian coordinates) are given by

(3) $$\frac{\partial u_{i}}{\partial x_{i}}=0,$$

(4) $$\rho \left(\frac{\partial u_{i}}{\partial t}+u_{k}\frac{\partial u_{i}}{\partial x_{k}}\right)=-\frac{\partial P}{\partial x_{i}}+\frac{\partial }{\partial x_{j}}\left(\mu \frac{\partial u_{i}}{\partial x_{j}}\right)+\frac{\partial T_{ij}}{\partial x_{j}},$$

where t is the time, $x_{i}$ the spatial coordinate in the i-th direction, $u_{i}$ is the velocity component, P is the pressure, and $T_{ij}$ is the Reynolds stress tensor component. The velocity components and the pressure in Equations (3) and (4) are time-averaged quantities. In addition, the k−ω Shear Stress Transport (SST) turbulence model by Menter [56,57] is adopted to simulate the turbulent flow in the dynamic filtration system, as used in previous studies on rotating disk filtration systems [31,32]. The k−ω SST model is a two-equation model, which simultaneously solves two transport equations related to the turbulence kinetic energy k and the specific dissipation rate ω. The fundamental idea of the turbulence model is to use the k−ω model in the boundary layer region, while activating the k−ε model outside the boundary layer, using a blending function. Then, the modification to the eddy viscosity is introduced.

When modeling flows due to rotating parts, in this study caused by a rotating patterned disk, the simulation methods using unsteady moving grids in the absolute reference frame are computationally expensive and cumbersome to use [58]. As an alternative to the moving grid methods, a multiple reference frame (MRF) method is a cost-effective way to analyze many engineering flows involving rotating parts, e.g., quadcopters, wind turbines, fans, mixing tanks, and turbine blades [59]. In this regard, an MRF method implemented in ANSYS CFX 18.1 (ANSYS Inc., Canonsburg, PA, USA) is used to solve the rotating flow in the dynamic filtration system. When using the MRF method, the computational domain is divided into two or more domains, which may be either rotating or stationary. When solving the governing equations for each domain, relevant data are exchanged at the interfaces between two different domains. After solving the governing equations, one can obtain the relative velocity in a moving reference frame, defined by

(5) $$u_{R}=u-\Omega \times r,$$

where $u_{R}$ is the relative velocity (the velocity viewed from a moving frame of reference), u the absolute velocity (the velocity viewed from a stationary frame of reference), Ω the angular velocity, and r the position vector.

The boundary conditions for solving the governing equations are considered as follows. At the inlet ($\Gamma_{in}$), a feed pressure of 100,100 Pa is imposed, and a pressure of 100,000 Pa is applied at the outlet ($\Gamma_{out}$). Thus, the pressure difference between the inlet and outlet is kept at 100 Pa. On the membrane ($\Gamma_{p}$), the permeate flux 𝒥 (the velocity normal to the membrane surface) is specified by

(6) J=u·n=KΔP,

where n is the outward unit normal vector, K the membrane permeability constant (in this study $K=5\times 10^{-10}\;m/s\cdot \mathrm{Pa}$), and ΔP the transmembrane pressure (TMP). The details on the membrane used in numerical simulations will be introduced in Section 3.5 when dealing with the filtration performance in our dynamic filtration module. In this study, assuming that the gauge pressure on the permeate side is zero, the TMP is the pressure at the membrane surface. No-slip condition is specified at the disk surface ($\Gamma_{r}$) and at the shaft surface ($\Gamma_{s}$). Thus, the circumferential velocity component $u_{\theta }$ on $\Gamma_{r}$ and $\Gamma_{s}$ (in the fixed coordinate) is $u_{\theta }=r\Omega$, where r the radial distance from the z-axis. At the impermeable boundaries ($\Gamma_{w}$), such as the housing walls, the no-slip and nonpenetration conditions are imposed, i.e., u=0 at $\Gamma_{w}$. 

### 2.3. Numerical Scheme

In this study, numerical simulations are conducted using commercial CFD software (ANSYS CFX 18.1), which is based on the finite volume method. The computational domains are discretized by hexahedral elements with the number of elements ranging from approximately 16,600,000 to 18,600,000 depending on the disk shape in the DF system. The number of elements is determined from a mesh convergence test, which will be introduced in Section 3.1. A high-resolution scheme is adopted to treat the convection terms in the governing equations, preventing numerical instabilities such as spurious oscillations or wiggles from occurring. As for solver, an algebraic multigrid (AMG) method is used in solving the governing equations in an iterative manner. All the computations have been conducted using parallel processing with 16 processors in a workstation with dual 16-core CPUs (Intel^®^ Xeon^®^ Gold 6130, 2.1 GHz) and 256 GB memory. The set of governing equations is solved using a convergence criterion with the RMS residuals less than $10^{-5}$.

## 3. Results and Discussion

This section begins with the results of a mesh convergence test that aimed to determine an optimal element size for the entirety of the simulations. Next, the flow characteristics induced by the rotating disk and the applied pressure difference between the inlet and outlet ports are introduced. In addition, the wall shear stress distribution on the membrane for each filtration system with a specific patterned disk is also evaluated. The shear stress-induced antifouling effect, closely related to the shear stress level at the membrane surface [51], is characterized by the area-averaged friction coefficient. Finally, the filtration performance is assessed using the permeate flux that is affected by both the disk shape and the disk rotating speed. 

### 3.1. Mesh Convergence Test and Validation

**Figure 3:**
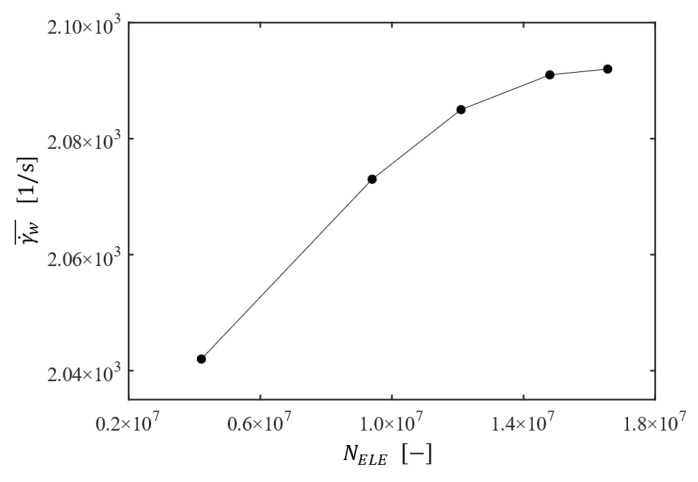
The change of the average shear strain rate $\bar{{\dot{\gamma }}_{w}}$ at the membrane wall as a function of the number of elements $N_{ELE}$ for the filtration system with the plain disk rotating at Ω=200 rpm.

First, the proper number of elements is determined through a mesh convergence test, guaranteeing a reliable solution with an affordable computational cost. For this purpose, numerical simulations for the filtration system with the plain disk (P00 in Figure 2) operating at Ω=200 rpm were conducted. The number of elements is varied from 4 to 20 million. In addition, the mesh is kept finer near the stator wall and the membrane surface to capture the large velocity gradients in the boundary layers. As for the convergence criterion, an RMS residual of $1\times 10^{-5}$ is required to ensure the convergence of solutions. The average shear strain rate ($\bar{{\dot{\gamma }}_{w}}$) at the membrane surface is employed as an indicator to check mesh convergence because it is sensitive to the velocity solution in the boundary layer. Figure 3 shows the change of the average wall shear strain rate as the number of elements ($N_{ELE}$) increases. The grid system with approximately 20 million elements, showing convergence with mesh refinement, is used in CFD simulations introduced in the following sections. 

**Figure 4:**
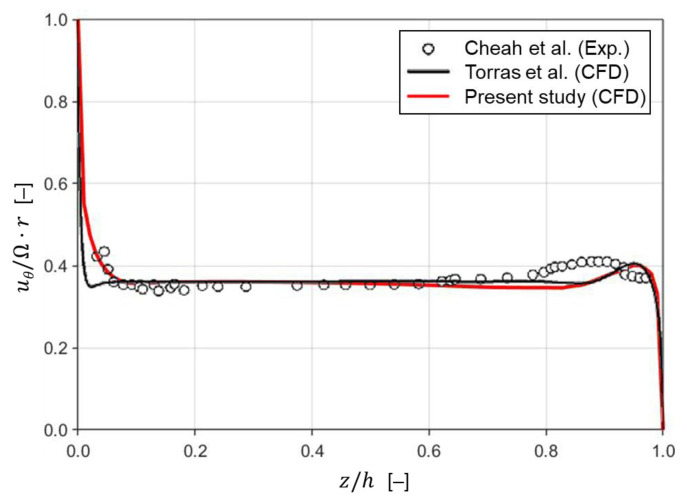
Comparison of the mean circumferential velocity ($u_{\theta }$) profiles at r/R=0.4 along the gap between the disk and the membrane surface when Re=1.6×106. The red line indicates the velocity profile obtained numerically in the present study. Both circles and the black line are velocity profiles in [31] (reprinted from Torras et al. [31] with permission from Elsevier). The original experimental data (circles) are from Cheah et al. [60].

To validate the numerical scheme used in this study, our velocity profile for an enclosed rotor-stator cavity flow with the aspect ratio 0.127 and the Reynolds number $1.6\times 10^{6}$ is compared with an experimental data reported by Cheah et al. [60]. The mesh for the cavity geometry used in the validation contains 2,880,000 hexahedral elements. Torras et al. [31] also compared their simulation results (obtained using commercial software Fluent 6.2) for the same geometry and operating condition with those in [60]. Figure 4 shows the time-averaged circumferential velocity ($u_{\theta }$) profiles at r/R=0.4 along the gap between the disk and the membrane surface for the three cases when Re=1.6×106. The red line is the velocity profile obtained from the present CFD simulation, while the circles and the black line are the velocity profiles from [60] and [31], respectively. Though the two CFD results show deviations from the experimental data near the stator surface (z/h=1), they are in a good agreement with the experimentally measured velocity profile. As for predicting the velocity profile near the disk surface (z/h=0), our CFD model predicts the experimental data better than that of [31], ensuring the accuracy of the present numerical scheme in predicting the flow characteristics of the rotor-stator cavity system. 

### 3.2. Flow Charactetistics

In the dynamic filtration (Figure 1), high wall shear stresses are created by the relative motion between the fixed membrane and a rotating patterned disk. Therefore, a better understanding of the in-depth flow characteristics is necessary to determine a proper set of design parameters and operating conditions resulting in an enhanced filtration performance and reduced membrane fouling. In what follows, all the velocity components are presented in terms of dimensionless variables. Here, the dimensionless radius ($r^{*})$, the dimensionless mean velocity components ($u_{\theta }^{*}$ and $u_{r}^{*}$), and the dimensionless axial coordinates $z^{*}$ are defined by $r^{*}=r/R_{i},$ $u_{\theta }^{*}=u_{\theta }/U$, $u_{r}^{*}=u_{r}/U$, and $z^{*}=z/H$, where $r=\sqrt{\left(x^{2}+y^{2}\right)}$, $U=R_{i}\Omega$ and $H=h_{g}+h_{d}/2$ (see Figure 1 for the geometrical parameters).

**Figure 5:**
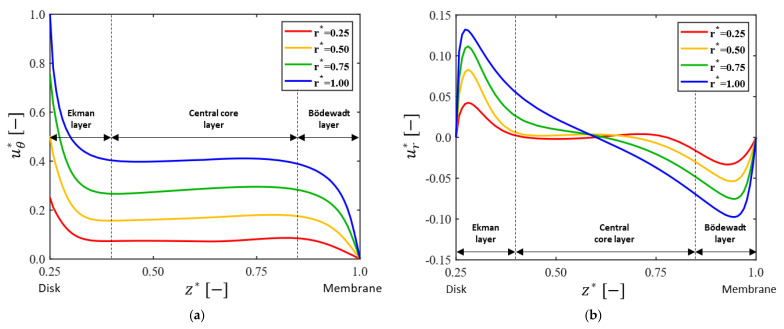
The dimensionless circumferential and radial velocity profiles for the plain disk case (P00). Velocity profiles in the dimensionless axial direction are plotted at the four locations on the y-axis with the dimensionless radial distance $r^{*}=$ 0.25, 0.5, 0.75, and 1. Here, the angular velocity of the disks is Ω=200 rpm. The two velocity components are defined by $u_{\theta }^{*}=u_{\theta }/U$ and $u_{r}^{*}=u_{r}/U$, where $U=R_{i}\Omega$. (**a**) Dimensionless circumferential velocity ($u_{\theta }^{*}$ ); (**b**) dimensionless radial velocity ($u_{r}^{*}$ ). In the two plots, $z^{*}=0.25$ corresponds to the disk surface and $z^{*}=1$ to the membrane surface.

First, the basic flow in the dynamic filtration system with a disk without pattern (the plain disk P00 in Figure 2) will be presented. Figure 5 depicts the dimensionless circumferential and radial velocity profiles ($u_{\theta }^{*}$ and $u_{r}^{*}$) in the dimensionless axial direction $z^{*}$ at the four radial positions $r^{*}=$ 0.25, 0.5, 0.75, and 1 (in this case located on the y-axis), when Ω=200 rpm (corresponding to the Reynolds number of Re=1.6×105). In Figure 5, the abscissa $z^{*}=0.25$ corresponds to the disk surface and $z^{*}=1$ to the membrane surface. The circumferential velocity profile (Figure 5a) clearly demonstrates the existence of three layers in the axial direction, a central core layer and two boundary layers separated by the core layer, which is a typical flow pattern in the regime IV of a rotor-stator cavity flow as defined by Daily and Nece [41]. This type of flow is called the Batchelor-type flow with three distinct zones: a centrifugal boundary layer close to the rotor (Ekman layer), a central core with negligible variation in $u_{\theta }^{*}$, and a centripetal boundary layer near the stator (Bödewadt layer). Though it is not presented in the paper, the axial velocity magnitude $\left|u_{z}^{*}\right|$ is order of $O\left(10^{-3}\right)$, and is thus quite small compared to the magnitude of the tangential and radial velocity components. The velocity gradients near the disk and membrane surfaces increase with $r^{*}$, indicating that the frictional effect on both surfaces increases in the radial direction as well.

**Figure 6:**
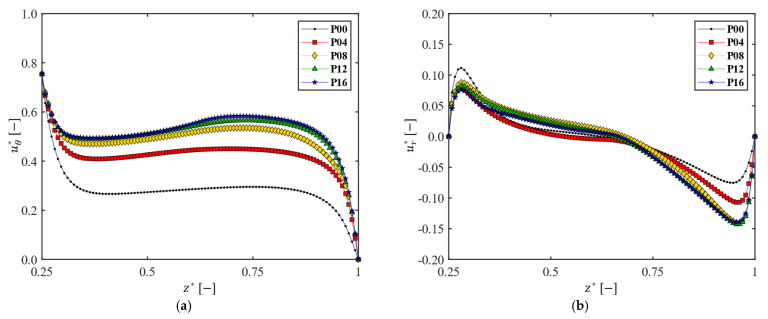
The influence of the number of patterns on the dimensionless circumferential and radial velocity profiles. The velocity profiles are measured at a location on the y-axis with the radial distance $r^{*}=$ 0.75, when Ω=200 rpm. (**a**) Dimensionless circumferential velocity ($u_{\theta }^{*}$ ); (**b**) dimensionless radial velocity ($u_{r}^{*})$.

Next, the influence of the number of patterns on the velocity profiles was investigated, varying the number of patterns $N_{p}$ from 0 to 16 with an increment of 4 (Figure 2). By inserting wheel-shaped patterns on disk surfaces, complex vortices are expected to occur, affecting the mean velocity profiles, the radial distribution of pressure, the shear stress on the wall, and the permeate flux. The velocity profiles for $u_{\theta }^{*}$ and $u_{r}^{*}$ affected by the number of patterns are depicted in Figure 6, where the velocity components ($u_{\theta }^{*}$ and $u_{r}^{*}$ in the axial direction) at $r^{*}=$ 0.75 are plotted. When patterns are engraved on the disk, the circumferential velocity magnitude in the central core region increases. It was reported that increasing the magnitude of the circumferential velocity in the central core region produces a higher permeate flux at the same angular velocity [61]. Thus, one can expect a higher permeate flux as the number of patterns increases in the filtration module. The flux enhancement with $N_{p}$ will be discussed in Section 3.5.

As the number of patterns $N_{p}$ increases, the magnitude of the velocity gradient $\partial u_{\theta }^{*}/\partial z^{*}$ decreases at the disk surface, but increases at the membrane surface (Figure 6a). The increase rate of the velocity gradient magnitude at the membrane surface (${\left|\partial u_{\theta }^{*}/\partial z^{*}\right|}_{z^{*}=1}$) tends to be saturated when $N_{p}>8$. In the case of a patterned disk, therefore, one can expect a higher shear stress on the membrane and better antifouling due to the higher wall shear stress, compared with those from a plain disk. The patterns on the rotating disk play a role in carrying the momentum caused by the disk rotation further into the central core region, leading to an increased velocity magnitude $\left|u_{\theta }^{*}\right|$ with $N_{p}$ in this region. As for the variation of $u_{r}^{*}$ with $N_{p}$, there exist changes in the velocity profile near both walls. The magnitude of the velocity gradient $\partial u_{r}^{*}/\partial z^{*}$ at the membrane surface increases with $N_{p}$, but it does not show any notable change as $N_{p}$ increases above 8, which is a similar trend to that observed in the change of $\partial u_{\theta }^{*}/\partial z^{*}$. However, the influence of such changes on the frictional effect are not as significant as those observed in $u_{\theta }^{*}$, since the magnitude of $u_{\theta }^{*}$ is one order of magnitude less than that of $u_{r}^{*}$ (Figure 6b). The quantitative analysis on the shear stress increase with the number of patterns will be discussed in Section 3.4.

**Figure 7:**
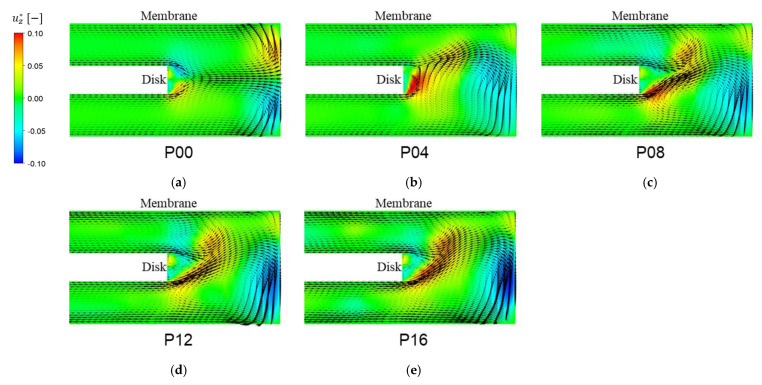
The cross-sectional velocity vectors (in the zx-plane) influenced by the number of patterns $N_{p}$ when Ω=200 rpm. The color contour in each plot represents the value of the dimensionless axial velocity ($u_{z}^{*}$ ) defined by $u_{z}^{*}=u_{z}/\left(\Omega R_{i}\right)$. (**a**) $N_{p}=0$ (plain disk), (**b**) $N_{p}=4$, (**c**) $N_{p}=8$, (**d**) $N_{p}=12$, and (**e**) $N_{p}=16$.

Figure 7 shows the cross-sectional velocity vectors and contours for the value of the dimensionless axial velocity $u_{z}^{*}$ in the zx-plane. As depicted in Figure 7a, the flow pattern for the plain disk (P00) looks almost symmetric with respect to the midsurface (xy-plane) even though there are small perturbations due to the pressure-driven flow effect. The two counter-rotating cross-sectional flows near the rim of the housing generate a pair of Taylor vortices. Vortex structures varying with $N_{p}$ will be presented in Section 3.5. However, such symmetry is broken completely for the cases with patterns on the disk. As the number of patterns increases above 8, one cannot observe notable changes in the cross-sectional velocity vectors from a qualitative perspective.

### 3.3. Vortex Structure

The performance of a rotary dynamic filtration system is closely related to a secondary flow, such as Taylor vortices created by a high-speed rotation of a rotor, reducing membrane fouling or concentration polarization [18]. Such a secondary flow in membrane filtration also leads to enhanced mixing and improved fouling resistance via back transport of foulants from the membrane to the bulk flow region [27,53]. In addition, Taylor vortices are also associated with the enhanced shear rate in a dynamic filtration module [61]. In this regard, three-dimensional vortex structures due to the flow in the DF system are identified using the velocity field. Among several definitions representing the topology of a vortex core, the $\lambda_{2}$-definition of Jeong and Hussain [62] is adopted for vortex identification in our filtration system. In this definition, a vortex core is defined as a connected fluid region with two negative eigenvalues of $D^{2}+W^{2}$. Here, D and W are the symmetric and antisymmetric parts of the velocity gradient tensor ∇u, respectively, where $D=\left(\nabla u+\nabla u^{T}\right)/2$, and $W=\left(\nabla u-\nabla u^{T}\right)/2$. If the three real eigenvalues ($\lambda_{1},\;\lambda_{2},$ and $\lambda_{3}$) of the symmetric tensor $D^{2}+W^{2}$ are ordered as follows: $\lambda_{1}\geq \lambda_{2}\geq \lambda_{3}$, the definition is equivalent to the requirement that $\lambda_{2}<0$ within the vortex core.

**Figure 8:**
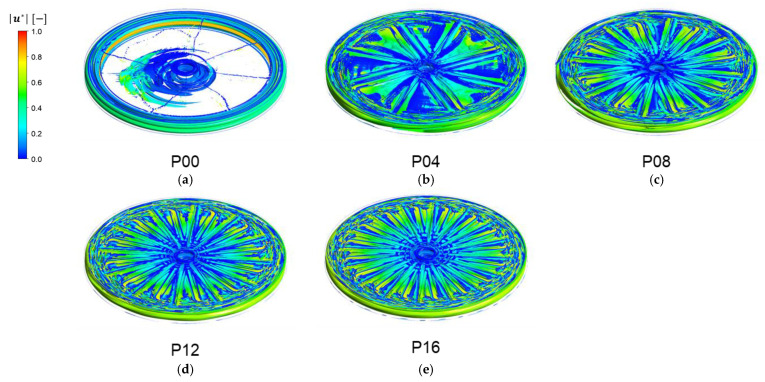
The vortex structures affected by the number of patterns $N_{p}$, visualized by $\lambda_{2}$ -criterion when the angular velocity of the disk is Ω=200 rpm. The color contour in each plot represents the dimensionless velocity magnitude ($\left|u^{*}\right|$ ). (**a**) $N_{p}=0$ (plain disk), (**b**) $N_{p}=4$, (**c**) $N_{p}=8$, (**d**) $N_{p}=12$, and (**e**) $N_{p}=16$.

As far as vortex structures are concerned, our main concern is on the change of vortex structure with the number of patterns on the disk. As representative examples, vortex structures at a fixed rotation speed (Ω=200 rpm) will be presented in this section. Figure 8 demonstrates the three-dimensional vortex structures using the $\lambda_{2}$-definition for the filtration modules with different number of patterns when the disk rotating speed is Ω=200 rpm. In this figure, vortex regions are identified by iso-surfaces of $\lambda_{2}$ and color contours represent the magnitude of the dimensionless velocity $u^{*}=u/U$. In the case of a plain disk (P00), a pair of Taylor vortices are observed in the radial gap between the disk and the outer housing (Figure 8a). The upper one represents rotating motions in the counter-clockwise direction and the lower one represents those in the clockwise direction, which are generated by the flow pattern depicted in Figure 7. In addition, vortices also emerge in the upper and lower axial gaps near the disk tip. Spiral vortices, skewed to the left (in the negative x-direction), occur near the center of the filtration module. The skewness in the spiral vortices is due to the existence of the outlet port located at the bottom of the housing (see Figure 1). Thin vortices, which are stretched in the radial direction, are observed near the stator wall. In the cases of filtration modules with a patterned disk, there exists a significant difference in the vortex structures.

As the number of patterns $N_{p}$ increases, the two Taylor vortices become distorted, showing a wavy pattern (Figure 8b), and they are merged together at the highest $N_{p}$ (Figure 8e). The evolution of the vortex structures with $N_{p}$, formed in the entire domain of the rotor-stator system, demonstrates the dependency of the vortex structures on the disk geometry. It should be noted again that, as the number of patterns $N_{p}$ increases above 8, the changes in the vortex structure in the radial gap between the disk and the housing begin to be saturated with $N_{p}$.

### 3.4. Shear Stress Distribution 

The flow and vortex structures presented in Section 3.2 and Section 3.3 revealed the number of patterns on the disk surface has a significant impact on the flow behavior in the dynamic filtration module. The rotational motions of the patterned disk can generate a high wall shear stress at the membrane surface as well, affected in this study by the number of patterns $N_{p}$ and the Reynolds number Re. In addition, the shear stress at the membrane is closely related to the control of membrane fouling and the filtration enhancement in dynamic filtration modules [25,26,27,30]. As such, we attempt to elucidate the variation of the wall shear stress at the membrane and its change with $N_{p}$ and Re.

The variation of the wall shear stress on the membrane was examined using the line-averaged friction coefficient $C_{f}$ at a radial position r, defined by

(7) $$C_{f}=\frac{1}{2\pi r}\int_{0}^{2\pi }\tau_{w}^{*}r\;d\theta ,\;$$

where the integrand is evaluated along a circle on the membrane surface with the radius *r* and its center at (x,y)=(0,0). In Equation (7), $\tau_{w}^{*}$ is the dimensionless wall shear stress on the membrane, defined by

(8) $$\tau_{w}^{*}=\frac{\tau_{w}}{\frac{1}{2}\rho \Omega^{2}R_{i}^{2}}.$$

The numerator $\tau_{w}$ in Equation (8) is the local wall shear stress at the membrane surface, given by $\tau_{w}=\mu {\dot{\gamma }}_{w}$, where μ is the viscosity, ${\dot{\gamma }}_{w}$ the wall shear rate (${\dot{\gamma }}_{w}={\sqrt{2D:D}|}_{w}$), and D the rate-of-deformation tensor (symmetric part of the velocity gradient tensor). The denominator in Equation (8) is the dynamic pressure defined with the characteristic velocity $\Omega R_{i}$ and the characteristics length $R_{i}$. In the Cartesian coordinate system, the shear rate $\dot{\gamma }$ is evaluated using the following relation:

(9) $$\dot{\gamma }=\sqrt{2\left\{{\left(\frac{\partial u}{\partial x}\right)}^{2}+{\left(\frac{\partial v}{\partial y}\right)}^{2}+{\left(\frac{\partial w}{\partial z}\right)}^{2}\right\}+{\left(\frac{\partial u}{\partial y}+\frac{\partial v}{\partial x}\right)}^{2}+{\left(\frac{\partial v}{\partial z}+\frac{\partial w}{\partial y}\right)}^{2}+{\left(\frac{\partial u}{\partial z}+\frac{\partial w}{\partial x}\right)}^{2}},$$

where u, v, and w are velocity components in the x–, y–, and z–directions, respectively.

**Figure 9:**
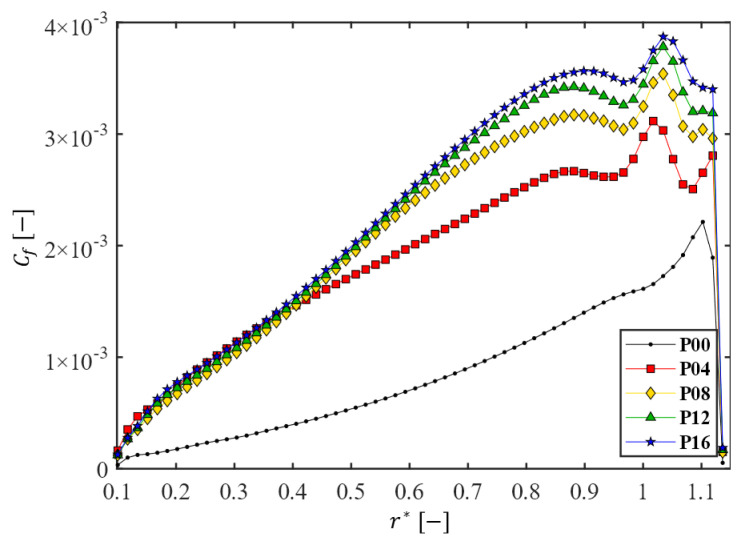
The change of the line-averaged friction coefficient $C_{f}$ at the membrane surface as a function of the dimensionless radius $r^{*}=r/R_{i}$ for the five filtration modules with different numbers of patterns on the disk. Here, Ω=200 rpm.

Figure 9 shows the change of the line-averaged friction coefficient $C_{f}$ at the membrane surface as a function of the dimensionless radius $r^{*}=r/R_{i}$ for the five filtration modules with different numbers of patterns ($N_{p}=0$, 4, 8, 12, and 16). Though the rotation speed is a factor with an influence on the shear stresses [63], the friction coefficient when Ω=200 rpm (equivalent to Re=1.6×105) is presented as a representative example. In the case of the plain disk with $N_{p}=0$ (P00), the friction coefficient $C_{f}$ increases with $r^{*}$, reaches a peak value around $r^{*}\approx 1.1$, and decreases rapidly near the stator side wall. Such variation of $C_{f}$ with $r^{*}$ can also be inferred from Figure 5 showing the velocity components ($u_{\theta }^{*}$ and $u_{r}^{*}$) and their changes in the z-direction. If wheel-shaped patterns are engraved on the disk surfaces, a different profile of $C_{f}$ is observed in each filtration module. In cases of disks with patterns, the overall values of $C_{f}$ increased significantly as $N_{p}$ increased, compared to the case with $N_{p}=0$ (P00). When $N_{p}=16$ (P16 in Figure 9), the maximum friction coefficient is approximately two times higher than that of the case with $N_{p}=0$, and the peak value is observed around $r^{*}\approx 1.03$.

**Figure 10:**
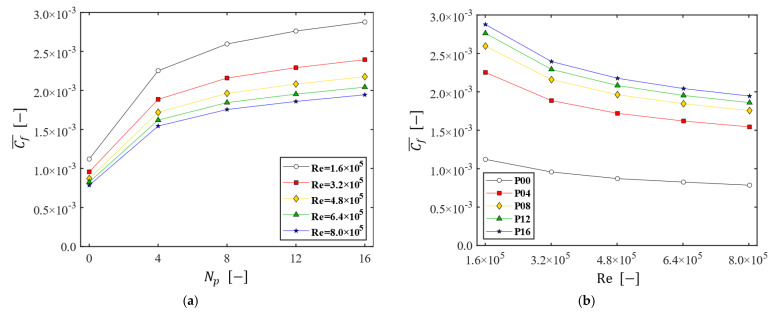
The change of the area-averaged friction coefficient ($\bar{C_{f}}$). (**a**) $\bar{C_{f}}$ as a function of the numbers of patterns $N_{p}$ at the five Reynolds numbers; (**b**) $\bar{C_{f}}$ as a function of the Reynolds number, affected by $N_{p}$.

The influence of the number of patterns ($N_{p}$) and the Reynolds number (Re) on the wall shear stress is investigated by utilizing a friction coefficient. To evaluate the influence of $N_{p}$ and Re on the skin friction over the entire membrane surface, the area-averaged friction coefficient $\bar{C_{f}}$ is defined as follows:

(10) $$\bar{C_{f}}=\frac{1}{A_{m}}\iint_{A}\tau_{w}^{*}\;dA,$$

where $A_{m}$ denotes the membrane area ($A_{m}=3.14\times 10^{-2}$ m^2^) and A the domain on the membrane. Figure 10 shows the change of the area-averaged friction coefficient ($\bar{C_{f}}$) influenced by $N_{p}$ and Re. Here, the disk rotating speed Ω is varied from 200 to 1000 rpm (thus, Re is varied from $1.6\times 10^{6}$ to $8.0\times 10^{6}$). Figure 10a plots $\bar{C_{f}}$ as a function of $N_{p}$ at the five Reynolds numbers, while Figure 10b plots $\bar{C_{f}}$ as a function of Re at the five values of $N_{p}$. As $N_{p}$ increases $\bar{C_{f}}$ increases as well, but the increase rate of $\bar{C_{f}}$ shows a noticeable slowdown when $N_{p}>8$, regardless of the Reynolds number (Figure 10a). As for the effect of Re on the skin friction, $\bar{C_{f}}$ decreases with Re and the decrease rate of $\bar{C_{f}}$ increases as $N_{p}$ increases (Figure 10b). Although $\bar{C_{f}}$ decreases with Re, the magnitude of $\tau_{w}$ itself increases with Re. In summary, the filtration dynamic module with a patterned disk leads to a higher friction coefficient than that of the plain disk and the increase rate of $\bar{C_{f}}$ with $N_{p}$ slows down as the number of patterns $N_{p}$ increases above 8.

### 3.5. Filtration Performance

Filtration enhancement due to the patterned disk in the dynamic filtration module is demonstrated for a microfiltration process. In a microfiltration membrane, the permeate flux J through the membrane is given by J=KΔP. Assuming that the membrane pores are well-connected, uniform, and cylindrical in shape, the permeability constant K [64] is evaluated using the following relation:

(11) $$K=\frac{\phi a^{2}}{8\mu \zeta \Delta l},$$

where ϕ is the porosity, a the pore radius, ζ the tortuosity, and Δl the membrane thickness. Using the following parameters for the membrane and the feed fluid, ϕ=0.2, a=0.1 μm, μ=0.001 Pa∙s, ζ=2.5, and Δl=200 μm, the permeability constant is $K=5\times 10^{-10}$ m/s·Pa. Though membrane fouling is a major problem in most membrane processes, the fixed value of K is assumed, limiting our interest to the flux enhancement due to the rotating patterned disk in the dynamic filtration system with an uncontaminated membrane. As mentioned in Section 2.2, the local pressure at a specific location on the membrane is used as ΔP, assuming that the gauge pressure in the permeate side is zero. 

The flux enhancement due to a patterned disk is characterized by a dimensionless permeate flux $J^{*}$,

(12) $$J^{*}=\frac{J}{J_{0}},$$

where $J_{0}$ is the reference flux, defined by $J_{0}=K\Delta P_{0}$. Using $\Delta P_{0}=100,000$ Pa (reference TMP), the reference flux is $J_{0}=180$ LMH (L/m^2^·h), which is equivalent to $5\times 10^{-5}$ m/s. The change of $J^{*}$ in the radial direction is assessed using a line-averaged dimensionless permeate flux $J_{L}^{*}$, given by

(13) $$J_{L}^{*}=\frac{1}{2\pi r}\int_{0}^{2\pi }J^{*}r\;d\theta ,\;$$

which is defined in an analogous manner to the definition of $C_{f}$ (Equation (7)). To evaluate overall filtration performance of the filtration system influenced by $N_{p}$ and Re, the area-averaged dimensionless permeate flux $\bar{J^{*}}$ is defined as follows:

(14) $$\bar{J^{*}}=\frac{1}{A_{m}}\iint_{A}J^{*}\;dA.$$

**Figure 11:**
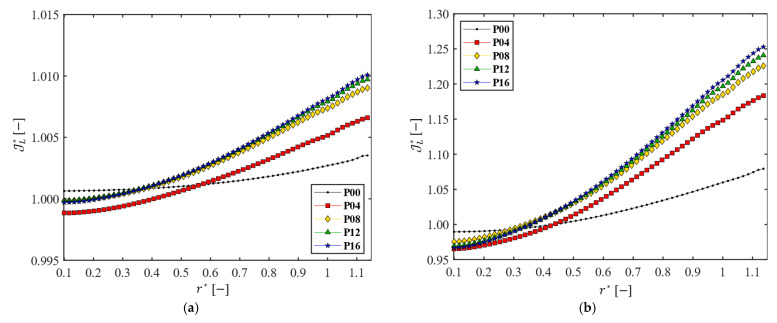
The line-averaged dimensionless permeate flux $J_{L}^{*}$ as functions of the dimensionless radius ($r^{*}=r/R_{i}$ ) for the five disks, when (**a**) Ω=200 rpm (Re=1.6×105 ) and (**b**) Ω=1000 rpm (Re=8.0×105 ).

Figure 11 shows the line-averaged dimensionless permeate flux $J_{L}^{*}$ as a function of the dimensionless radial coordinate $r^{*}$ for the five filtration modules with a different number of patterns. In all cases, $J_{L}^{*}$ increases with $r^{*}$ because the pressure at the membrane (TMP) increases in the radial direction. When a patterned disk is used, $J_{L}^{*}$ near the center is lower, compared with that of the module with the plain disk, but $J_{L}^{*}$ becomes higher in the outer portion of the membrane, bringing benefit in the permeate flux. As the number of patterns $N_{p}$ increases, the increase of $J_{L}^{*}$ is significant up to the number of patterns $N_{p}=8$, and it tends to be saturated when $N_{p}>8$. When Ω=200 rpm (Re=1.6×105), the highest flux obtained with $N_{p}=16$ is $J_{L}^{*}\approx 1.01$ (equivalent to 182 LMH), which is not a significant gain in the permeate flux. When Ω=1000 rpm (Re=8.0×105), however, the highest flux obtained with $N_{p}=16$ is $J_{L}^{*}\approx 1.25$, now equivalent to 225 LMH. The variation of $J_{ℒ}^{*}$ in the radial direction confirms that the effectiveness of the DF module is closely related to both the rotating speed of the patterned disk and the number of patterns.

**Figure 12:**
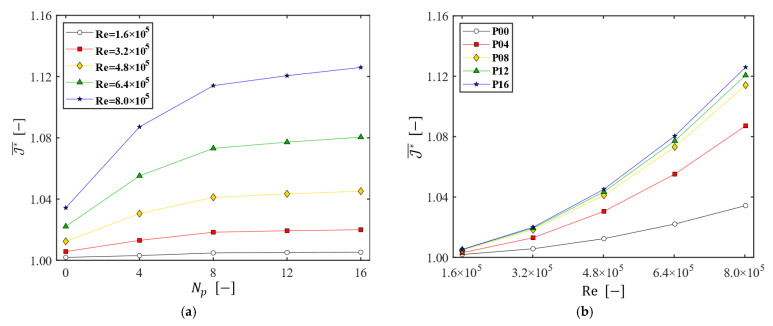
The area-averaged permeate flux ($\bar{J^{*}}$). (**a**) $\bar{J^{*}}$ as a function of $N_{p}$ at the five Reynolds numbers; (**b**) $\bar{J^{*}}$ as a function of Re for the five disks.

Finally, the overall flux enhancement with the patterned disk was assessed using the area-averaged permeate flux ($\bar{J^{*}}$), depicted in Figure 12. Within the range of $N_{p}$ and Ω used in this study, the maximum area-averaged permeate flux ${\bar{J^{*}}|}_{max}$ (obtained with $N_{p}=16$ and Re=8.0×105) is approximately 1.13 (equivalent to 203 LMH). The proper number of patterns seems to be eight, since one cannot achieve a notable gain in $\bar{J^{*}}$ when $N_{p}>8$. As for the effect of Re on $\bar{J^{*}}$, the higher the Reynolds number, the larger the gain in the permeate flux caused by the use of a patterned disk.

## 4. Conclusions

In this study, the effect of disk geometry on the permeate flux enhancement in a dynamic filtration (DF) system is numerically investigated. The DF system consists of a disk (rotor) and a stator with a membrane fixed on top of the stator. Wheel-shaped patterns are engraved on both sides of a disk with a number of patterns ($N_{p}$) on one side varying from 0 to 16. The aspect ratio G, the axial clearance (between the disk and the membrane) divided by the disk radius, is fixed at 0.0511. A constant pressure difference of 100 Pa is maintained between the inlet and outlet. The Reynolds number (Re) is varied from $1.6\times 10^{5}$ to $8.0\times 10^{5}$ by changing the disk rotating speed (Ω) from 200 to 1000 rpm. The k−ω SST turbulence model is employed to solve the turbulent rotating flows and the MRF method is adopted to treat the rotating disk in quasi-steady state.

The flow characteristics in the DF system are examined to understand the detailed flow affected by $N_{p}$ and Re. The velocity profiles along the axial direction revealed the Batchelor-type flow with two boundary layers (near the disk and membrane surfaces) and a central core layer in all Reynolds numbers. For a constant angular velocity, as the number of patterns $N_{p}$ increases, the magnitude of the circumferential velocity in the central core layer increases, which not only increases the shear stress applied to the membrane surface but also improves the permeate flux. The vortex structure in the dynamic filtration system is visualized via the $\lambda_{2}$-criterion, demonstrating complex vortex structures generated by the perturbation due to the patterned surfaces. The changes of flow characteristics (velocity profiles, shear stresses, and vortex structures) and the overall permeate flux tend to be saturated as $N_{p}$ increases above 8, implying that $N_{p}=8$ is a proper choice for the number of patterns in the DF system. Within the range of $N_{p}$ and Ω investigated in this study, the maximum area-averaged permeate flux obtained when $N_{p}=16$ and Re=8.0×105 is approximately 203 LMH, while the flux for the plain disk at the same Re is approximately 185 LMH. It should be noted that a rotating disk with a proper number of patterns resulted in a higher wall shear stress, mitigating membrane fouling and a higher permeate flux compared to that from a filtration system with a plain disk, but the gain in the permeate flux is highly affected by the Reynolds number (or the disk rotating speed Ω). The higher the Reynolds number, the larger the gain in the permeate flux from a patterned disk in the DF system. 

As a fundamental study on the hydrodynamics in a rotor-stator-type DF system, this study presents new insights into the effect of the pattered disk on the flow and filtration performance in the DF system that have not been previously explored. In future work, we plan to investigate the concentration distribution and permeate flux enhancement in a reverse-osmosis DF system with patterned disks, and to solve the associated flow and mass transfer problems.

## Data Availability

The data supporting the findings of this study are available from the corresponding author upon reasonable request.

## Abbreviations

AMG	Algebraic multigrid
CFD	Computational fluid dynamics
CFF	Crossflow filtration
CP	Concentration polarization
DF	Aynamic filtration
MRF	Multiple reference frame
MSD	Multishift disk
RANS	Reynolds-averaged Navier-Stokes
RMS	Root mean square
SST	Shear stress transport
SWM	spiral-wound module
TMP	transmembrane pressure
**List of Symbols**	
$A_{m}$	Membrane area, m^2^
a	Pore radius of membrane, μm
$C_{f}$	Friction coefficient
$\bar{C_{f}}$	Area-averaged friction coefficient
D	Symmetric part of velocity gradient, 1/s
G	Aspect ratio
H	Gap from the x–axis to membrane, mm
$h_{g}$	Axial gap, mm
J	Permeate flux, m/s
$J_{0}$	Reference permeate flux, m/s
$J^{*}$	Dimensionless permeate flux
$\bar{J^{*}}$	Area-averaged dimensionless permeate flux
K	Membrane permeability, m/s·Pa
k	Turbulence kinetic energy, J/kg
l	Membrane thickness, μm
$N_{ELE}$	Number of elements
$N_{p}$	Number of patterns on one side of a disk
n	Outward unit normal vector
P	Pressure, Pa
$P_{\mathrm{in}}$	Pressure at the inlet, Pa
$P_{\mathrm{out}}$	Pressure at the outlet, Pa
R	Radius, mm
$R_{i}$	Disk radius, mm
$R_{o}$	Stator radius, mm
$R_{s}$	Shaft radius, mm
Re	Reynolds number
r	Radial distance from the z–axis, mm
$r^{*}$	Dimensionless radius
t	Time, s
$T_{ij}$	Reynolds stress tensor, Pa
U	Linear velocity, m/s
u	Velocity vector, m/s
$u_{R}$	Relative velocity vector, m/s
$u_{\theta }$	Circumferential velocity, m/s
$u_{r}$	Radial velocity, m/s
$u_{z}$	Axial velocity, m/s
$u^{*}$	Dimensionless velocity vector
$u_{\theta }^{*}$	Dimensionless circumferential velocity
$u_{r}^{*}$	Dimensionless radial velocity
$u_{z}^{*}$	Dimensionless axial velocity
W	Antisymmetric part of velocity gradient, 1/s
$z^{*}$	Dimensionless z–coordinate
**Greek Letters**	
$\Gamma_{in}$	Inlet boundary
$\Gamma_{out}$	Outlet boundary
$\Gamma_{p}$	Membrane (permeable boundary)
$\Gamma_{r}$	Disk surface
$\Gamma_{s}$	Shaft surface
$\Gamma_{w}$	Impermeable boundary
$\dot{\gamma }$	Shear rate, 1/s
${\dot{\gamma }}_{w}$	Wall shear rate, 1/s
$\bar{{\dot{\gamma }}_{w}}$	Average wall shear rate, 1/s
Δ	Difference
ζ	Tortuosity
λ	Real eigenvalue
μ	Viscosity of fluid, Pa·s
ρ	Density of fluid, kg/m^3^
$\tau_{w}$	Wall shear stress, Pa
$\tau_{w}^{*}$	Dimensionless wall shear stress
ϕ	Porosity
Ω	Angular velocity, 1/s
ω	Specific dissipation rate, 1/s
